# Fusimotor control of spindle sensitivity regulates central and peripheral coding of joint angles

**DOI:** 10.3389/fncom.2012.00066

**Published:** 2012-08-30

**Authors:** Ning Lan, Xin He

**Affiliations:** ^1^School of Biomedical Engineering, Med-X Research Institute, Shanghai Jiao Tong UniversityShanghai, China; ^2^School of Dentistry, Division of Biokinesiology and Physical Therapy, University of Southern CaliforniaLos Angeles, CA, USA

**Keywords:** muscle spindle, γ_s_ control, spindle sensitivity, *Ia* afferents, joint angle, central and peripheral coding

## Abstract

Proprioceptive afferents from muscle spindles encode information about peripheral joint movements for the central nervous system (CNS). The sensitivity of muscle spindle is nonlinearly dependent on the activation of gamma (γ) motoneurons in the spinal cord that receives inputs from the motor cortex. How fusimotor control of spindle sensitivity affects proprioceptive coding of joint position is not clear. Furthermore, what information is carried in the fusimotor signal from the motor cortex to the muscle spindle is largely unknown. In this study, we addressed the issue of communication between the central and peripheral sensorimotor systems using a computational approach based on the virtual arm (VA) model. In simulation experiments within the operational range of joint movements, the gamma static commands (γ_s_) to the spindles of both mono-articular and bi-articular muscles were hypothesized (1) to remain constant, (2) to be modulated with joint angles linearly, and (3) to be modulated with joint angles nonlinearly. Simulation results revealed a nonlinear landscape of *Ia* afferent with respect to both γ_s_ activation and joint angle. Among the three hypotheses, the constant and linear strategies did not yield *Ia* responses that matched the experimental data, and therefore, were rejected as plausible strategies of spindle sensitivity control. However, if γ_s_ commands were quadratically modulated with joint angles, a robust linear relation between *Ia* afferents and joint angles could be obtained in both mono-articular and bi-articular muscles. With the quadratic strategy of spindle sensitivity control, γ_s_ commands may serve as the CNS outputs that inform the periphery of central coding of joint angles. The results suggest that the information of joint angles may be communicated between the CNS and muscles via the descending γ_s_ efferent and *Ia* afferent signals.

## Introduction

Muscle spindle is a unique sensory organ that has dual efferent and afferent innervations (Boyd, 1980; Matthews, 1981; Hulliger, 1984). A large amount of cortical outputs is directed to γ motoneurons that supply fusimotor control of spindles (Boyd and Smith, 1984). A larger number of studies have been dedicated to elucidate the morphological, biochemical, and neurophysiological properties of the spindle (Matthews, 1962; Granit, 1970; Boyd and Smith, 1984). But relatively little has been revealed about the functional role of fusimotor efferent in the execution of motor tasks, because it has been difficult, if not impossible, to record directly from gamma motor neurons during normal movements. Fusimotor control is so far best understood to adjust the sensitivity of muscle spindles. As the alpha motor neurons activate extrafusal muscle fibers to produce a contraction force, the spindle is unloaded. To keep the spindle sensitive during muscle contraction, the central nervous system (CNS) may co-activate the intrafusal fiber via descending gamma commands γ (Vallbo and al-Falahe, 1990), in order to assess the outcome of the alpha activation of muscles. In so doing, if γ_s_ command were properly modulated with movement, the spindle firing may not be interrupted by the unloading effects of muscle contraction. Early studies have associated the spindle function to regulation of muscle length (Merton, 1953; Stein, 1974; Houk and Rymer, 1981). But difficulties of the length-servo hypothesis have turned the direction of research towards more centrally organized programming for motor control (Flash and Hogan, 1985; Feldman, 1986; Hasan, 1986; Corcos et al., 1989; Gottlieb et al., 1989; Uno et al., 1989; Harris and Wolpert, 1998; Todorov and Jordan, 2002). On the other hand, central programming or coding of sensorimotor control must take into account the peripheral constraints presented in the neuromuscular system (Kawato et al., 1990; Lan and Crago, 1994; Lan, 1997). Thus, it is necessary to elucidate the nature of information communicated between the central and peripheral systems.

It has been a main subject of experimental studies with regard to the nature of gamma fusimotor commands relevant to motor control (Boyd, 1980; Matthews, 1981; Hulliger, 1984; Boyd et al., 1985). Only until recently, experimental studies of patterns of gamma motor activity during movement and posture in animals have shed some light to the plausible function of fusimotor co-activation with α commands (Taylor et al., 2004). Direct recordings from gamma fibers in reduced cat preparations showed that there was in-phase modulation of γ_s_ activities with muscle EMGs during locomotion, providing firm evidence of α –γ co-activation during movement (Taylor et al., 2000). And static gamma activity was considered to be a fusimotor template of intended movement (Taylor et al., 2006). This implied that γ_s_ signal might carry centrally planned kinematic information of joint angles. In the periphery, direct recording of *Ia* afferents from the dorsal ganglion cells of decerebrated cats indicated a robust linear relation between *Ia* afferents and joint angles (Stein et al., 2004). In human subjects, direct recording of spindle afferents from the extensor carpi radialis brevis (ECRb) and extensor digitorum (ED) (Cordo et al., 2002) revealed that the steady-state population firing of *Ia* afferents was found linearly related to joint position during the hold period between ramps. In these experiments, γ_s_ modulation of spindle sensitivity was unknown in both animal and human recordings. However, the evidence in reduced animal preparations and intact human subjects provided partial clues on the central and peripheral coding of joint positions by fusimotor (γ_s_) commands and *Ia* afferent signals.

In a more theoretical approach, a number of studies have suggested that trajectory and final position of movement may be planned separately, and executed with a dual control strategy (Lan et al., 2005; Ghez et al., 2007; Scheidt and Ghez, 2007). Experimental evidence also indicated that the brain treats movement and position information with distinct neural representations (Kurtzer et al., 2005). Injection of the γ-aminobutyric acid (GABA) antagonist picrotoxin into cat's reticular part of the substantia nigra (SNR) removed static fusimotor action from spindle primary endings (Wand and Schwarz, 1985). On the other hand, electrical stimulation at fasciculus retroflexus region of the cat's midbrain reproduced dynamic fusimotor effect, indicating that the habenulo-interpeduncular system may be involved in generating dynamic gamma commands (Taylor and Donga, 1989). Thus, movement and position control signals may be generated and processed in different regions of the brain, and passed down to spinal motor neurons as separate descending commands (Lemon, 2008). A set of static commands may be most relevant to maintaining a steady state limb position (Lan et al., 2005), while a set of dynamic commands may control dynamic acceleration and deceleration of movements (Lan and Crago, 1994; Lan, 1997; Lan et al., 2005). In this framework of dual control, it is necessary that the CNS inform the peripheral neuromuscular system about the desired joint position via a pathway separate from the α commands to the muscles. Recent experimental data (Cordo et al., 2002; Taylor et al., 2006) imply that an alternative pathway for transmission of kinematic information is via γ commands to muscle spindles.

In this study, we used a computational approach to explore the functional role of fusimotor system in transmitting the centrally planned joint kinematics to the periphery, and how a robust linear relation between *Ia* afferent and joint angle could be achieved with fusimotor control of spindle sensitivity. With a computational model of the virtual arm (VA) (Song et al., 2008a; He et al., 2012), we tested three plausible strategies of fusimotor control of spindle sensitivity with constant, linear and nonlinear modulations with joint angles. The correlation between joint angles and *Ia* afferents under different fusimotor control strategies were investigated for mono-articular and bi-articular muscles. The hypotheses were rejected or accepted based on the consistence of simulated behaviors to those of experiments. Part of the preliminary results was presented in a conference proceeding (He and Lan, 2011).

## Materials and methods

### The sensorimotor systems model

**Figure 1:**
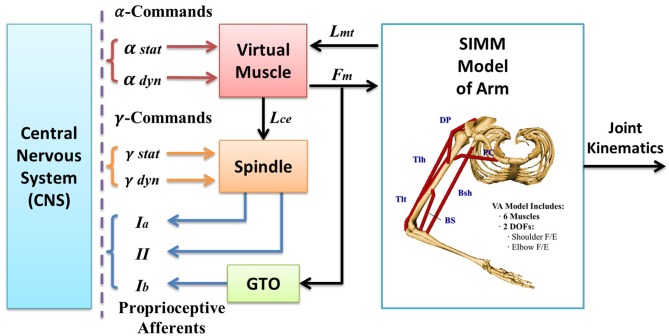
**The virtual arm (VA) model is an integrated neuromuscular sensorimotor systems model in SIMULINK, which encompasses an anatomically accurate structure of upper arm, physiologically realistic muscle mechanics and dynamics, and spindle and Golgi tendon organ (GTO) proprioceptors.** Each subcomponent embodies a set of mathematical equations obtained from previous experimental data in literature that describe the physiological, geometrical, kinematic, and dynamic properties of the subsystems. The VA model receives α and γ commands from the central nervous system (CNS), and outputs numerical results of simulation for all state variables, including joint kinematics and proprioceptive afferents (i.e., *Ia, Ib*, and *II* afferents). The biomechanical model of the VA has two degrees of freedom (DOFs) in horizontal plane (shoulder flexion/extension, elbow flexion/extension) and is driven by six muscles, which are clavicle portion of pectorailis major (PC) and deltoid posterior (DP) for shoulder joint, brachialis (BS) and triceps lateral head (Tlt) for elbow joint, and biceps short head (Bsh) and tricps long head (Tlh) cross both joints. The virtual muscle (VM) model activated by commands calculates contraction forces (*F*_m_) and instantaneous muscle fascicle length (*L*_ce_). The muscle spindle model receives inputs of fascicle length (*L*_ce_) and fusimotor modulation (γ_s_, γ_d_) and generates primary *(la)* and secondary *(II)* afferents. However, since we are interested in neural coding for joint angles in this study, only γ_s_ and *Ia* afferent signal is of interest in the simulation and analysis.

The computational model of the integrated, multi-joint sensorimotor VA system used in this study was shown in Figure 1. This model has been developed and validated for simulation studies of neural control of human arm movements (Song et al., 2008a; He et al., 2012). The VA model was capable of generating *Ia* afferents of muscles at different joint angles and under different fusimotor inputs. Thus, it was suitable to address the issue of how fusimotor control affects the coding of joint angles by *Ia* afferents. For completeness, a succinct description of the systems model was given below.

The VA systems model in Figure 1 was a two-joint arm in the horizontal plane. It consisted of subcomponent models of an anatomically accurate upper arm with shoulder and elbow joints, and physiologically realistic muscles and proprioceptors. Each model component has been validated respectively during its development (Cheng et al., 2000; Mileusnic et al., 2006; Song et al., 2008a,b), and then integrated into the realistic VA systems model in SIMULINK (Figure 1).

Computational modules of the VA model were implemented with a graphic modeling software SIMM and SIMULINK, respectively. The mathematical equations of geometry, kinematic, and dynamics of the multi-body system of the upper arm were embodied into SIMM, and the SIMM model was converted into a computational block in SIMULINK that computed joint motion with given muscular forces acting upon the joints (Song et al., 2008a). There were six representative muscles acting on the joints. The virtual muscle (VM) model contained all mathematical equations that described realistic muscle physiology and mechanics (Cheng et al., 2000), and a new version of the VM model was implemented in SIMULINK (Song et al., 2008b). The VM module computed muscle force and muscle fascicle length with given neural input after a continuous recruitment scheme (Song et al., 2008b). The new VM model improved computational efficiency and simulation stability. It allowed a continuous recruitment of slow and fast fibers, and decoupled α, γ command inputs to active extrafusal and intrafusal fibers, respectively.

Three pairs of agonist and antagonist muscles were selected to actuate two degrees of freedom (DOF) of the VA model in horizontal plane (Figure 1). Pectoralis major (clavicle portion, PC) and Deltoid posterior (DP) were mono-articular flexor and extensor at the shoulder joint; brachialis (BS) and triceps brachii lateral head (Tlt) were mono-articular flexor and extensor of the elbow joint; biceps brachii short head (Bsh) and triceps brachii long head (Tlh) were the bi-articular muscles cross both joints.

Each muscle model was embedded with a spindle model (Mileusnic et al., 2006) and a simplified Galgi tendon organ (GTO) model (Song et al., 2008a). The spindle model contained a bag1, a bag2, and a chain fiber with *Ia* and *II* afferent outputs. *Ia* afferents were sum of all fiber outputs and *II* afferents were primarily from chain fibers. The gamma static efferent innervated bag2 and chain fibers, and the gamma dynamic efferent innervated primarily bag1 fiber. Thus, the spindle model was capable of simulating spindle *Ia* and *II* responses to both static and dynamic fusimotor inputs.

### Determination of a set of equilibrium positions in simulation

**Figure 2:**
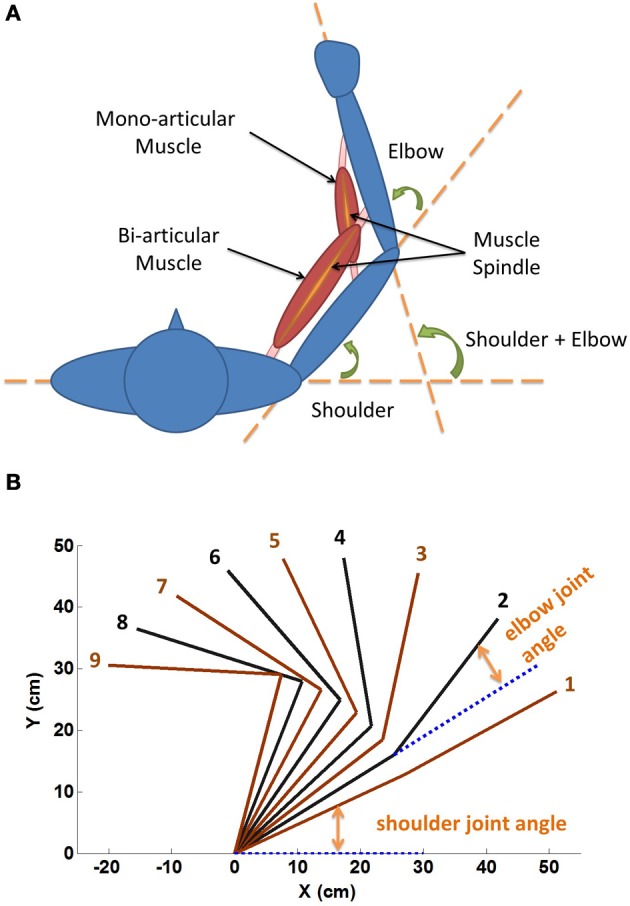
**(A)** Geometric definition of shoulder and elbow angles. The range of shoulder flexion is set from 0° (fully extended) to 120° (fully flexed), and the range of elbow flexion is from 0° (fully extended) to 150° (fully flexed). Showing in the figure are a typical mono-articular muscle crossing the elbow joint, and a typical bi-articular muscle crossing both shoulder and elbow joints. The spindles are arranged in parallel with muscle fascicle fibers. We hypothesize that muscle fascicle length (*L*_ce_) and *Ia* afferent are related to the corresponding joint angles of span. Thus for bi-articular muscles, they are related to the sum of joint angles of span. **(B)** Nine sets of α_stat_ commands (Table 1) are used to stabilize the VA model at nine equilibrium positions (1 ~ 9) in horizontal plane, respectively. At each position the spindle sensitivity control by γ_stat_ is investigated.

The definitions of shoulder and elbow angles were shown in Figure 2A. The range of shoulder flexion was set from 0° (fully extended) to 120° (fully flexed), and the range of elbow flexion was from 0° (fully extended) to 150° (fully flexed). Showing in Figure 2A were a typical mono-articular muscle crossing the elbow joint, and a typical bi-articular muscle crossing both shoulder and elbow. The spindles were arranged in parallel with muscle fascicle fibers. In this study, however, joint angles of shoulder and elbow were varied in the operational range within the full range of motion (ROM), as shown in Figure 2B.

A procedure of initialization for dynamic simulation used in (He et al., 2012) was adopted in this study to obtain a set of equilibrium positions as shown in Figure 2B. The α commands of the nine stable equilibrium positions were tabulated in Table 1. The procedure was effective to determine initial system parameters, such as, fascicle length and joint angles, so that simulation could converge and the shoulder and elbow joints could be stabilized to a desired equilibrium position. In each simulation, the total running time was about 30 (s), in which the initial 10 s were designed to allow simulation to converge. A random, signal dependent noise (SDN) (Jones et al., 2002) was added to the muscle activation (He et al., 2012) at about 10 (s) to reproduce the inherent variability in the neuromuscular system. The steady state joint angles and *Ia* afferents were calculated as the average value of data in the last 10 s of simulation.

**Table 1:** **Alpha static activation levels at nine positions**.

**Muscle**	**Position**								
	**1**	**2**	**3**	**4**	**5**	**6**	**7**	**8**	**9**
PC	0.25	0.30	0.35	0.40	0.45	0.50	0.55	0.60	0.65
DP	0.65	0.60	0.55	0.50	0.45	0.40	0.35	0.30	0.25
Bsh	0	0	0	0	0	0	0	0	0
Tlh	0	0	0	0	0	0	0	0	0
BS	0.25	0.30	0.35	0.40	0.45	0.50	0.55	0.60	0.65
Tlt	0.65	0.60	0.55	0.50	0.45	0.40	0.35	0.30	0.25

**Figure 3:**
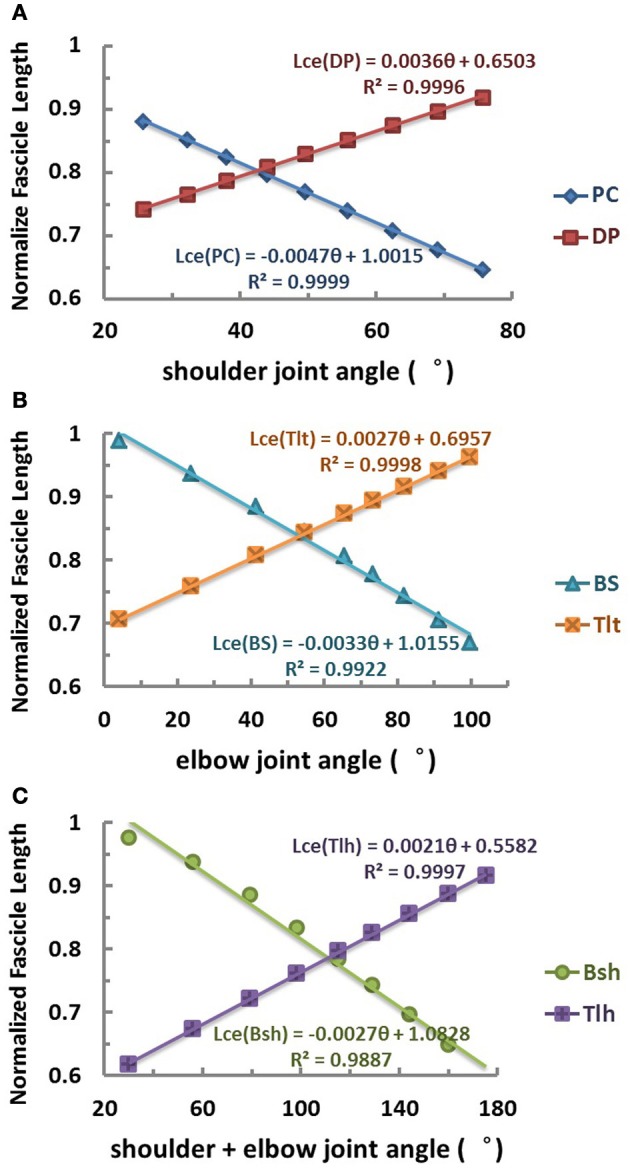
**Relation between equilibrium joint angle and muscle fascicle length (θ_EP_ − *L*_ce_)of each muscle obtained in the range of joint angles used in simulation. (A)** Relation of shoulder mono-articular muscles PC and DP. **(B)** Relation of elbow mono-articular muscles BS and Tlt. **(C)** Relation of bi-articular muscles Bsh and Tlh cross both shoulder and elbow joints. Results indicate that a nearly linear relation exists between muscle fibre length and joint angle for both mono-articular muscles and bi-articular muscles, because of the their arrangement. The fascicle length of flexor is shortened and that of extensor is lengthened with increase of the joint angles of span. For bi-articular muscles Bsh and Tlh, their fascicle length, *L*_ce_, is found linearly related to the sum of shoulder and elbow angles. The linearity in the geometric relations provides supportive evidence for a simple coding relationship between joint angles and spindle input and output that are related to muscle fascicle length.

At the set of equilibrium positions, the geometric relationships between joint angle and muscle fascicle length in all muscles was evaluated. This was one of the peripheral constraints for the central programming of control of both intrafusal and extrafusal fibers. The joints of the VA were placed to different angles in the workspace (Figure 2B) by choosing particular patterns of mono-articular muscle activations (Table 1). The musculotendon lengths of the muscles were calculated from the VA model and the corresponding fascicle lengths were obtained at these joint angles. The relationship between joint angles and muscle fascicle length in the operational range of joint movement was then assessed in Figure 3.

### Evaluation of spindle sensitivity

**Figure 4:**
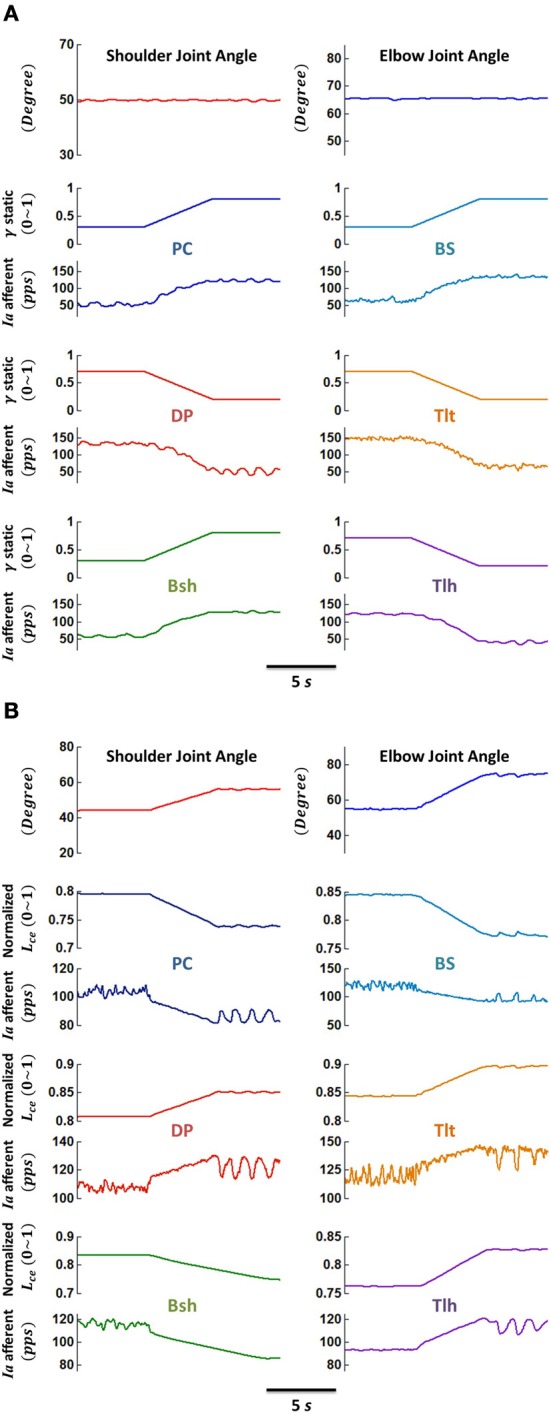
**Responses of primary afferents *(Ia)* of muscle spindles to (A) ramped γ_s_ drive, and (B) ramped joint angle (fascicle length) change, respectively. (A)** The VA was maintained at position 5, while γ_s_ commands of flexors ramped from 0.3 up to 0.8 and those of extensors ramped from 0.7 down to 0.2, concurrently within 5 s. The *Ia* afferents of all muscles were shown to be modulated in-phase with γ_s_ changes. **(B)** The VA was moved from position 4 to 6 within 5 s by ramped alpha commands of single joint muscles, while the γ_s_ commands of each muscle remained constant at 0.5. The muscle fascicle length changed simultaneously with joint angles, and the *Ia* afferents were modulated in-phase with changes in fascicle length *L*_ce_. These demonstrate the sensitivity *Ia* afferents with respect to fusimotor activation γ_s_ and muscle fascicle length *L*_ce_.

In this study, we focused on the effects of gamma static, γ_s_, control of spindle sensitivity with respect to muscle fascicle length change, while the gamma dynamic control was fixed to a constant level. An example of influences of gamma static control and joint angles on spindle sensitivity was explored in the six muscles. First, at a fixed joint configuration, the gamma static commands to all muscles were varied in a ramp pattern, and the *Ia* afferents of the six spindles showed simultaneous variation with the ramp change of the gamma static command. Then, the sensitivity of *Ia* afferent to fascicle length change was examined in response to ramp changes in joint angles with constant levels of gamma static inputs in the six muscles. These results were shown in Figure 4, and they verified the *Ia* sensitivity to both gamma static control and joint angle (or fascicle length).

**Figure 5:**
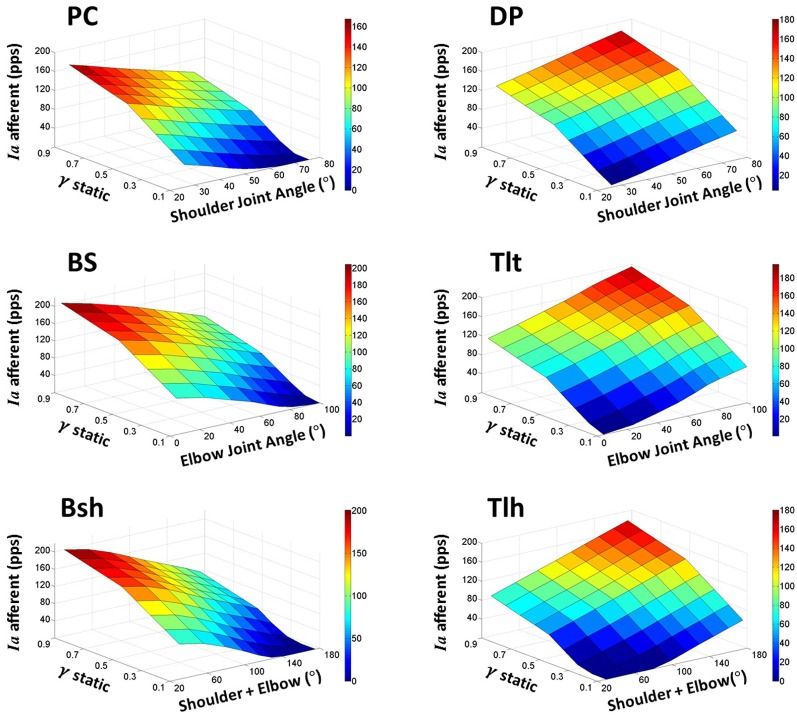
**The landscape of *Ia* afferent sensitivity with respect to the full ranges of fusimotor commands and muscle fascicle lengths (θ_EP_ − γ_s_ - *Ia*).** This is obtained by increasing fusimotor drive at each joint angle for all muscles incrementally. The sensitivity landscapes clearly reveal the complex interrelations of *Ia* afferents with both fusimotor control and joint angles. In general, the interrelation is nonlinear, and the nonlinearity is more prominent at the lower and higher values of fusimotor commands and joint angles. These nonlinearities may be due to sluggish sensitivity in the short fascicle length and saturation in the long fascicle length. However, it is also clear that the nonlinearity exists in the middle range of fusimotor commands for all muscles. This phenomenon reflects the nonlinear nature of physiological responses of muscle spindles.

The landscape of spindle *Ia* sensitivity with respect to joint angles and gamma static control was then further evaluated. In these simulations, alpha (α_s_) commands represented the activation level of motor neuron pool as inputs to the VA model. Each set of constant motor commands (α_s_, γ_s_) produced an equilibrium position of the arm with *Ia* afferents from six muscles. A total of nine sets of alpha commands to the shoulder and elbow muscles (Table 1) positioned the VA model to nine different equilibrium angles in the shoulder and elbow joints (Figure 2B). The gamma static (γ_s_) commands were changed from 0.0 to 1.0 with an increment of 0.1 at each of the joint angles. A total of 81 points in the θ_EP_ − γ_s_ − *Ia* space formed the landscape surface of *Ia* sensitivity for each muscle (Figure 5), which revealed the fundamental relationship among the three variables.

### Test of fusimotor control strategies

Three sets of simulation experiments were designed to evaluate the plausible strategies regarding spindle sensitivity control. Based on the shape of the sensitivity landscape in the θ_EP_ − γ_s_ − *Ia* space, fusimotor control strategies, represented by the relation between equilibrium angle and gamma static command (γ_s_ − θ_EP_), were hypothesized (1) to remain constant for all joint angles (H1); (2) to be modulated linearly with joint angles (H2); and (3) to be modulated quadratically with joint angles (H3). The resultant relation between *Ia* afferents and joint angles was evaluated under the three hypotheses of fusimotor control, and the outcome relation of the (*Ia* − θ_EP_) curve was compared to experimentally observed behaviors. The necessary condition to reject a hypothesis was that the outcome relation of *Ia* afferents with joint angles must be a linear relation (Cordo et al., 2002; Stein et al., 2004). But to form a sufficient set of conditions to accept a hypothesis, other physiological constraints of experimental evidence in addition to the linear (*Ia* −θ_EP_) output must be considered.

## Results

### The length-angle relation of muscles

The relation between muscle fascicle length and joint angle revealed a geometric constraint in the VA model. The geometric relation was characterized by the θ_EP_ − *L*_ce_ curves shown in Figure 3 for the six muscles. In the operational range of shoulder and elbow joints, simulation results showed that the fascicle length of both mono-articular and bi-articular muscles was linearly related to the joint angles they cross. For mono-articular muscles, the joint of span was either shoulder joint or elbow joint, and thus the fascicle length of mono-articular muscles was linearly proportional to either shoulder angle or elbow angle (Figures 3A,B). For bi-articular muscles, the joints of span were both shoulder and elbow joints, and thus the fascicle length was linearly proportional to the sum of shoulder and elbow angles (Figure 3C). It is worth to note that while this fitted relation was near linear within the operational range of joints, significant nonlinearity may occur at the two extremes of ROM of joints, because the wrap around curvature of the joint was most effective within the operational ROM. Thus, at the extreme of joint angles, the CNS may rely on additional modality of proprioception, such as joint receptors, to estimate the value of joints accurately.

### Spindle responses to changes in gamma static and fascicle length

Figure 4 illustrated that gamma fusimotor control and fascicle length changes can modulate the sensitivity of *Ia* afferents effectively. The primary (*Ia*) afferents of muscle spindles responded to ramped gamma static drive (Figure 4A) and ramped fascicle length (Figure 4B) differently. In the simulations for fusimotor modulation effects, the VA was stabilized at position 5, and the gamma static commands of flexors were ramped from 0.3 up to 0.8 and those of extensors from 0.7 down to 0.2 during a period of 5 s. It was shown that the *Ia* afferents of all muscles were modulated in proportion with gamma static changes (Figure 4A), showing a strong modulation of *Ia* sensitivity by gamma static commands. In the simulation for joint angle changes, the VA was moved from position 4 to 6 during a period of 5 s. Joint angles were ramped from initial position to destination with linear changes in alpha drives to single joint muscles, while gamma static commands of each muscle remained constant at 0.5. Figure 4B showed the response of the *Ia* afferents to joint angle (or muscle fascicle length) changes. Clearly, there was a speed sensitivity component in the *Ia* response that was not present in isometric gamma static sensitivity of Figure 4A. After reaching to the destination of joint position, the spindle outputs were generally settled to a new level, but were affected by the dynamics in the spindle model (Mileusnic et al., 2006), as well as noise in the neuromuscular system (Jones et al., 2002). In this case, the fascicle length variation was about 10%, and the *Ia* outputs at steady state were varied approximately with the similar percentage, indicating effective modulation of *Ia* sensitivity by joint angles (or fascicle length).

### The landscape of spindle sensitivity

In order to obtain a general view of spindle *Ia* afferents in the normal range of joint angles and with all possible gamma static values, we searched the joint angle—fusimotor command—primary afferent (θ_EP_ − γ_s_ − *Ia*) space. In these simulations, the joint angles were fixed to one of the nine positions in Figure 2B. Then the gamma static inputs to the six muscle spindles were varied from 0.1 to 1.0, and *Ia* afferents of the six muscle spindles were examined. The results of each muscle were plotted in the 3D graphs in Figure 5. It was shown in general that the response of *Ia* afferents was not linear with respect to either joint angles or fusimotor commands in the whole space. This was not surprise because of the nonlinear response of spindles to both fusimotor commands and fascicle length change. Nonlinearity mainly occurred at the shorter muscle length, where the intrafusal fibres were relaxed at lower gamma activation levels. On the other hand, saturation of *Ia* afferents at longer muscle length with higher gamma activation levels also gave rise to nonlinear spindle sensitivity. The landscape of *Ia* sensitivity depicted the interrelation between gamma static commands and joint angles that may dictate the fusimotor control strategy.

### Plausible fusimotor control strategies

If joint angle is coded in the descending γ_s_ commands in the CNS, the γ_s_ should be formulated as a function of joint angle, so that the resultant relation between *Ia* afferents and joint angles matches to that of experimentally observed linear relation. Thus, the central strategy of joint angle coding must take into account of the spindle sensitivity with respect to joint angles and fusimotor activation in Figure 5. Since the CNS has the luxury to control spindle sensitivity by fusimotor commands, the CNS may modulate spindle sensitivity by adjusting the central pattern of coding of joint angles, in order to keep the peripheral coding of joint angle by *Ia* afferents consistent and reliable under all conditions. The modulation of γ_s_ command may occur in a relatively narrow region, for example a constant γ_s_ value; or in a wide range of γ_s_ command between 0.0 and 1.0 in order to achieve a better resolution of coding. In this study, we examined three scenarios (hypotheses) regarding fusimotor control strategy: (1) γ_s_ was maintained constant within the range of joint angle; (2) γ_s_ was modulated with joint angle in a linear function of joint angle; and (3) γ_s_ was modulated in a nonlinear function with joint angles.

**Figure 6:**
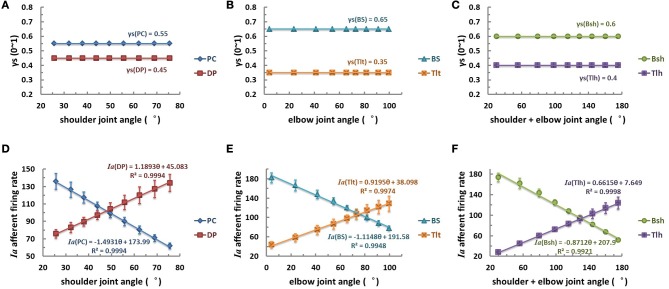
**The constant fusimotor control strategy, θ_EP_ − γ_s_, curves (A–C), and *Ia* afferents response (θ_EP_ − *Ia*) curves of muscle spindles (D–F), for each muscle.** The **(A,D)** is for shoulder actuators, the **(B,E)** is for elbow actuators, and the **(C,F)** is for bi-articular muscles. The abscissas indicate joint flexion angles while the vertical axis indicate activation levels **(A–C)** or firing rates **(D–F)**. The γ_s_ inputs of muscle spindles are set to medium constant levels at different angular positions, the firing rates of *Ia* afferents show an excellent linear relation with joint angles (with a goodness of fitting *R*^2^ > 0.99). This result may be evident from the sensitivity landscape of Figure 5, from which a constant γ_s_ value results in a fairly linear (θ_EP_ − *Ia*) relation.

The first scenario was tested by postulating that γ_s_ commands remained constant at modest activation levels within the entire range of joint angle θ (Figures 6A–C). The primary afferents of mono-articular muscle spindles showed a good linear relationship with corresponding joint angles (Figures 6D,E). The *Ia* afferents of bi-articular muscle spindles were also linearly proportional to the sum of shoulder and elbow joints as well (Figure 6F). The *Ia* afferents of extensor DP, Tlt, and Tlh were positively proportional to the joint angles, and *Ia* afferents of flexor PC, BS, and Bsh were negatively proportional to joint angles (Table 2). However, it was clear from Figure 5 that such good linear relations in θ_EP_ − *Ia* were only possible from median to high levels of γ_s_ activations. A constant fusimotor control may not be a physiological strategy because this does not allow any central coding of joint angles with γ_s_. In addition, experimental evidence did not support constant γ_s_ commands during movements (Taylor et al., 2000, 2004, 2006).

**Table 2:** **Fitting coefficients of constant γ_s_ strategy**.

	**γ_s_ = aθ^2^+bθ+c**			**Ia = kθ + e**
	**a**	**b**	**c**	**k**	**e**	**R^2^**
PC	0	0	0.55	−1.4931	173.99	0.9994
DP	0	0	0.45	1.1893	45.083	0.9994
BS	0	0	0.65	−1.1148	191.58	0.9948
Tlt	0	0	0.35	0.9195	38.098	0.9974
Bsh	0	0	0.60	−0.8712	207.90	0.9921
Tlh	0	0	0.40	0.6615	7.6490	0.9998
Average		–		1.0416 ± 0.2644		0.9972
				(pps/°)		

**Figure 7:**
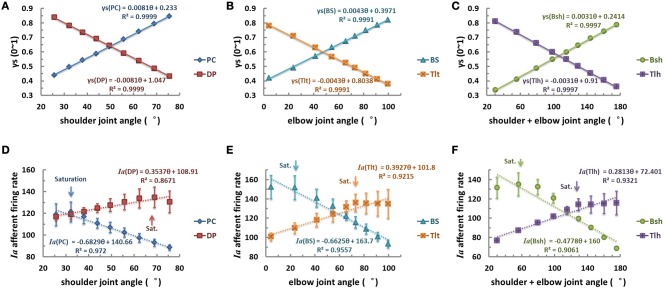
**The linear fusimotor control strategy, θ_EP_ − γ_s_ curves (A–C), and *Ia* afferents response, (θ_EP_ − *Ia*) curves of muscle spindles (D–F), for all muscles.** The **(A,D)** is for shoulder actuators, the **(B,E)** is for elbow actuators, and the **(C,F)** is for bi-articular muscles. The axes were defined the same as in Figure 6. When γ_s_ inputs of muscle spindles are linearly modulated with joint angles from 0.3 to 0.9, the firing rates of *Ia* afferents do not display a linear and monotonical relation to joint angles. Saturations of *Ia* responses occur at each muscle, as indicated by the arrows. This is due to the nonlinear physiological properties of muscle spindle demonstrated in Figure 5, and would not be possible to avoid if the full range of fusimotor commands were to be used to encode joint angle information.

The second scenario was then examined with γ_s_ varied linearly with joint angle θ (Figures 7A–C). The linear relation of θ_EP_ − γ_s_ could be learned by the CNS to specify desired position of joints. However, the results of *Ia* afferents of both mono-articular and bi-articular muscle spindles were not linearly related to joint angles, as was shown in Figures 7D–F. This nonlinear response was evident from the nonlinear landscape of *Ia* sensitivity shown in Figure 5, where nonlinearity occurred in the lower and higher regions of gamma activation and joint angles. As a result, there was saturation in the *Ia* response as indicated by the arrows in Figure 7. Thus, the outcome of the linear hypothesis of fusimotor control strategy did not give rise to a linear relation in θ_EP_ − *Ia* that was observed in experiments (Cordo et al., 2002; Stein et al., 2004). Therefore, the hypothesis of linear central coding of joint angle by γ_s_ command was rejected as a plausible strategy of fusimotor control.

**Figure 8:**
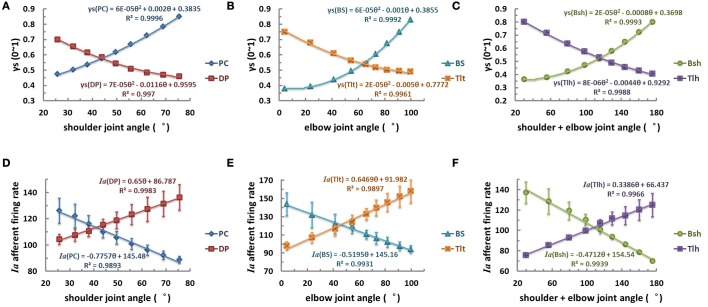
**The quadratic strategy of fusimotor control, θ_EP_ − γ_s_ curves (A–C), and *Ia* afferents response, (θ_EP_ − *Ia*) curves of muscle spindles (D–F), for each muscle.** The **(A,D)** is for shoulder actuators, the **(B,E)** is for elbow actuators, and the **(C,F)** is for bi-articular muscles. The axes were defined the same as in Figure 6. When γ_s_ inputs of muscle spindles are modulated with joint angles θ_*EP*_ quadratically in a monotonical manner with joint angles from 0.3 to 0.9, the firing rates of *Ia* afferents show a robust linear relation with joint angles with an average goodness of linear fitting *R*^2^ > 0.99. This phenomenon suggests that it is possible to manipulate the fusimotor commands to linearize the nonlinear *Ia* sensitivity revealed in Figure 5.

Consequently, this led us to consider the third scenario of a nonlinear monotonic coding of joint angle by fusimotor command, which would yield a linear output in *Ia* afferents with respect to joint angles. We hypothesized that γ_s_ commands were modulated quandratically with joint angle θ in parabolic curves of θ_EP_ − γ_s_, as shown in Figures 8A–C for the six muscles. Results showed that the *Ia* afferents of the six muscles were well correlated with joint angles linearly, as shown in Figures 8D–F, with a goodness of fitting *R*^2^ > 0.99. The outcome of this strategy appeared to fit all experimental data available (Cordo et al., 2002; Stein et al., 2004), and thus it may be the most likely strategy that the CNS may adopt for fusimotor control of spindle sensitivity. Note that although the central coding relationship between θ_EP_ − γ_s_ is non-linear, it remains a monotonic curve. This implies that γ_s_ could encode joint position uniquely within the ROM of joints. This phenomenon suggests that it is possible to manipulate the fusimotor commands to linearize the nonlinear *Ia* sensitivity revealed in Figure 5. The coefficients of fitted equations are presented in Table 3, which may be used in future simulation studies.

**Table 3:** **Fitting coefficients of quadratic γ_s_ strategy**.

	**γ_s_ = aθ^2^ + bθ + c**			**Ia = kθ + e**			
	**a**	**b**	**c**	**R^2^**	**k**	**e**	**R^2^**
PC	6e-5	0.0020	0.3835	0.9996	−0.7757	145.48	0.9893
DP	7e-5	−0.0116	0.9595	0.9970	0.6500	86.787	0.9983
BS	6e-5	−0.0010	0.3855	0.9992	−0.5195	145.16	0.9931
Tlt	2e-5	−0.0050	0.7772	0.9961	0.6469	91.982	0.9897
Bsh	2e-5	−0.0008	0.3698	0.9993	−0.4712	154.54	0.9939
Tlh	8e-6	−0.0044	0.9292	0.9988	0.3386	66.437	0.9966
Average		–		0.9983	0.5670 ± 0.1417		0.9935
					(pps/°)		

## Discussion

The understanding of neural control of movement would not be complete without revelation of the nature of fusimotor control for intrafusal fibers. The presence of enormous efferent and afferent innervations in the spindle prompts the question of what functional implications this sophisticated system may have, given that there is a vast CNS neural circuitry dedicated to process efferent and afferent neural information. The afferent signals may provide the CNS with peripheral kinematic information that allows the CNS to assess the outcome of executed motor action. But it is not straightforward as to what information content is carried in the efferent fusimotor signals to the spindle in the periphery. A more compelling fact is that there are more neurons in the motor cortex that innervate spinal gamma motoneurons than those that control alpha motoneurons (Boyd and Smith, 1984). Thus, it is imperative to understand the significance of the large amount of corticospinal outflows of fusimotor control to the peripheral musculoskeletal system.

There have been early efforts to identify indirectly the profiles of fusimotor control signals during motor performance (Hulliger and Prochazka, 1983; Hulliger et al., 1987). This was largely conducted with *Ia* afferent data recorded from reduced preparations of passively behaving animal models. An inverse simulation method was proposed to deduce the fusimotor profile from recorded *Ia* afferents along with movements. However, it was then realized that the fusimotor profile could be entirely different in voluntarily behaving animals from those in reduced animal models. Also, there were intermediate variables, such muscle fascicle length, musculotendon length and joint angle, that may all affect the accuracy of estimates. A more stringent limit was due to the nonlinear dynamics of the musculoskeletal responses, which may lead to a non-unique pattern of fusimotor activity, even though optimization technique may help reduce the uncertainty of estimation.

In spite of technical difficulty, recently, direct recording from gamma motor neurons in reduced animal models had successfully produced considerable insight into the fusimotor activation profiles during movements (Taylor et al., 2000, 2004, 2006). It was observed that static gamma activity formed a fusimotor template of intended movement (Taylor et al., 2006), and may carry kinematic information of joint angles. In the periphery, direct recording of *Ia* afferents from the dorsal ganglion cells of decerebrated cats suggested a similar conclusion that *Ia* afferents carried joint angle information (Stein et al., 2004). Similar conclusion was confirmed in human subjects with a voluntary contraction task (Cordo et al., 2002), which revealed that the steady-state population firing of *Ia* afferents was found linearly related to joint position during the hold period between ramps. Notice that in the experiments of Stein et al. (2004) and Cordo et al. (2002), the γ_s_ modulation of spindle sensitivity was unknown. But separate recordings of fusimotor activities and *Ia* afferents suggested that fusimotor efferents may be programmed in the CNS such that *Ia* afferents reliably inform the kinematics of peripheral limb movements. More importantly, these experimental results formed a set of necessary conditions that simulated behaviors of muscle spindle apparatus must conform. In the sense of mathematical proof using computational methods, a hypothetical fusimotor control strategy must reproduce all experimentally observed behaviors simultaneously. Strategies that do not simultaneously satisfy these necessary conditions could not be considered biologically plausible.

With this criterion in mind, we used a computational approach to address these related issues, (1) what specific kinematic information is encoded in gamma static fusimotor efferents? And (2) how gamma static command may be controlled in order to maintain a consistent *Ia* encoding of joint angles during movements. The peripheral factors that may affect the outcome of central coding were examined first with the VA model in Figures 3–5. It was interesting to note that the variability caused by the internal SDN noise simplified the relation between muscle fascicle length and joint angles in certain degree to a proximately linear relation within the operating range of joint angles, because of the averaging effects as shown in Figure 3. This appeared to lessen the nonlinear effects in the peripheral musculoskeletal system. However, the spindle sensitivity did show a significant nonlinear response that could affect both central and peripheral coding of joint angles (Figure 5). With prominent nonlinear spindle response, three hypotheses were tested regarding the central coding strategies of joint angles. The first set of simulation rejected the hypothesis that a constant γ_s_ control may be a plausible neural strategy in spite of its excellent linear θ_EP_ − *Ia* relation. Experimental evidence clearly showed that a dynamic pattern of γ_s_ modulation was observed to co-vary with α command and joint angles during movements (Taylor et al., 2000, 2004, 2006). In the test of second hypothesis of this study, the linear θ_EP_ − γ_s_ modulation did not produce a well-regulated linear θ_EP_ − *Ia* relation, which was observed in experimental recordings in man and animals (Cordo et al., 2002; Stein et al., 2004). This outcome may be attributable to the nonlinear sensitivity presented in the spindle response in Figure 5. Thus, the hypothesis of linear control strategy of spindle sensitivity expressed by linear θ_EP_ − γ_s_ correlation was also rejected. Then, we tested the third hypothesis, in which a nonlinear control strategy of γ_s_ commands may avoid the nonlinear zone in the landscape of spindle sensitivity in Figure 5. The results indicated that a second order nonlinear relation between γ_s_ command and joint angle, i.e., a parabolic θ _EP_ − γ_s_ curve, was indeed necessary, in order for the *Ia* afferent to be linearly correlated with joint angle. With the quadratic strategy, the *Ia* afferents of both mono-articular and bi-articular muscles displayed the similar property of a robust linear relation with joint angles. This result implies that the brain could learn the peripheral constraints, and program the nonlinear central coding of the monotonic θ _EP_ − γ_s_ curve, and send the γ_s_ command to inform the periphery about the centrally planned joint angles. Such central coding strategy would also allow the spindle to maintain an accurate and consistent encoding of angular information in *Ia* afferents, which the brain needs to evaluate the peripheral performance.

Lastly, we calculated the average position sensitivity of *Ia* afferents of all six muscles from simulation results for the three strategies of fusimotor control. The slopes of the linear θ_EP_ − *Ia* relationship were compared to that of Cordo's et al. (2002) human physiological recordings. The average position sensitivity under constant gamma control was 1.04 ± 0.29 pps/° (Table 2), which was much higher than that of quadratic strategy of gamma modulation of 0.57 ± 0.16 pps/° (Table 3). The latter was closer to that of position sensitivity of holding rate measured by Cordo et al. (2002), which was 0.40 ± 0.30 pps/°. This further supports the quadratic hypothesis as a plausible fusimotor control strategy for postures and movements.

The implication that fusimotor signals encode kimematic information of planned (or desired) movements is consistent with the finding that fusimotor activities were enhanced when performing a naïve task (Hulliger, 1984). When a new movement is performed, the CNS needs to program an optimal pattern of kinematics that is represented in fusimotor commands. This may necessitates modifying the planned kinematics frequently from practice to practice. The heightened activities seen in the fusimotor signals suggest that a process of searching for optimal kinematics is going on for the new task. In this process, proprioceptive afferents are used to assess the outcome of motor action, and are compared to the centrally programmed kinematics to detect any deviations between the programmed movement and outcome movement. Modifications are made in both centrally planned kinematics (gamma commands) and motor actions (alpha commands) to further optimize movement performance. Thus, the motor learning and control system acts like a reference adaptive control system, where the gamma commands provide the reference trajectories of movement (Taylor et al., 2006), and the alpha commands produce optimal driving inputs to the extrafusal muscle fibers. To fully appreciate the reference adaptive control of movement, central encoding of dynamic fusimotor commands with respect to movement kinematics should also be elucidated in the future research.

## Conclusion

We examined the peripheral factors that may influence the central and peripheral coding of joint angles through efferent and afferent innervations of the spindle apparatus. Based on these peripheral constraints, we have tested three hypotheses regarding static fusimotor control strategies of mono-articular and bi-articular muscles to achieve a reliable encoding and decoding of joint angle information. Results suggest that a quadratic strategy of static fusimotor control could lead to a linear relation between *Ia* afferents and joint angle with an average sensitivity close to the experimental value. This suggests that the γ_s_ command encodes joint position information in the CNS with a parabolic θ_EP_ − γ_s_ curve. Under the strategy of quadratic γ_s_ control, the peripheral linear θ_EP_ − *Ia* relation could be maintained, and used to decode actual angular information reliably from *Ia* afferents.

### Conflict of interest statement

The authors declare that the research was conducted in the absence of any commercial or financial relationships that could be construed as a potential conflict of interest.

## Appendix

### List of acronyms and symbols

α—Alpha motoneurons
α_d_, α_dyn_—Alpha dynamic command
α_s_, α_stat_—Alpha static command
BS—Brachialis
Bsh—Biceps Brachii short head
γ—Gamma motoneurons
γ_d_, γ_dyn_—Gamma dynamic command
γ_s_, γ_stat_—Gamma static command
CNS—Central Nervous System
DOF—Degree of freedom
DP—Deltoid Posterior
ECRb—Extensor Carpi Radialis brevis
ED—Extensor Digitorum
EMG—Electromyography
θ_EP_—Equilibrium point angle
*F*_m_—Muscle force
GABA—γ-aminobutyric acid
GTO—Golgi Tendon Organ
*Ia*—Primary afferent from spindle
*Ib*—afferent from GTO
*II*—Secondary afferent from spindle
*L*_ce_—Muscle fascicle length
*L*_mt_—Musculo-tendon length
PC—Pectoralis major Clavicle portion
pps—pulse per second
ROM—Range of Motion
SDN—Signal Dependent Noise
SNR—Substantia Nigra
Tlh—Triceps Brachii long head
Tlt—Triceps Brachii lateral head
VA—Virtual Arm
VM—Virtual Muscle
